# Functional neural correlates of reduced physiological falls risk

**DOI:** 10.1186/1744-9081-7-37

**Published:** 2011-08-16

**Authors:** Lindsay S Nagamatsu, Chun Liang Hsu, Todd C Handy, Teresa Liu-Ambrose

**Affiliations:** 1 Department of Psychology, University of British Columbia, 2136 West Mall, Vancouver BC, V6T 1Z4, Canada; 2 Centre for Hip Health and Mobility, Vancouver Coastal Research Institute, University of British Columbia, 7/F 2635 Laurel Street, Vancouver BC, V6H 2K2, Canada; 3 Brain Research Centre, University of British Columbia, 2211 Wesbrook Mall, Vancouver BC, V6T 2B5, Canada; 4 Department of Physical Therapy, University of British Columbia, #212 2177 Wesbrook Mall, Vancouver BC, V6T 1Z3, Canada

## Abstract

**Background:**

It is currently unclear whether the function of brain regions associated with executive cognitive processing are independently associated with reduced physiological falls risk. If these are related, it would suggest that the development of interventions targeted at improving executive neurocognitive function would be an effective new approach for reducing physiological falls risk in seniors.

**Methods:**

We performed a secondary analysis of 73 community-dwelling senior women aged 65 to 75 years old who participated in a 12-month randomized controlled trial of resistance training. Functional MRI data were acquired while participants performed a modified Eriksen Flanker Task - a task of selective attention and conflict resolution. Brain volumes were obtained using MRI. Falls risk was assessed using the Physiological Profile Assessment (PPA).

**Results:**

After accounting for baseline age, experimental group, baseline PPA score, and total baseline white matter brain volume, baseline activation in the left frontal orbital cortex extending towards the insula was negatively associated with reduced physiological falls risk over the 12-month period. In contrast, baseline activation in the paracingulate gyrus extending towards the anterior cingulate gyrus was positively associated with reduced physiological falls risk.

**Conclusions:**

Baseline activation levels of brain regions underlying response inhibition and selective attention were independently associated with reduced physiological falls risk. This suggests that falls prevention strategies may be facilitated by incorporating intervention components - such as aerobic exercise - that are specifically designed to induce neurocognitive plasticity.

**Trial Registration:**

ClinicalTrials.gov Identifier: NCT00426881

## Introduction

Falls are a major health care problem for seniors and health care systems. They are the third leading cause of chronic disability worldwide [1] and approximately 30% of community-dwellers over the age of 65 years experience one or more falls every year [2]. Importantly, 5% of falls result in fracture, with one-third of those being hip fractures.

Key risk factors for falls include reduced physiological function, such as impaired balance, [3,4] and cognitive impairment [2]. Recent evidence suggests that even mild reductions in cognitive abilities among otherwise healthy community-dwelling older adults increase physiological falls risk [5-8]. Specifically, evidence suggests that reduced *executive functions *-- the ability to concentrate, to attend selectively, and to plan and strategize -- are associated with increased falls risk among seniors without cognitive impairment and dementia [5,6,9-11].

Currently, the neural basis for the association between reduced executive functions and falls is unclear. Evidence from neuroimaging studies provides insight to possible underlying mechanisms. Specifically, cerebral white matter lesions (or leukoaraiosis) are associated with both reduced executive functions [12] and gait and balance abnormalities [13-16]. Cerebral white matter lesions may interrupt frontal lobe circuits responsible for normal gait and balance or they may interfere with long loop reflexes mediated by deep white matter sensory and motor tracts [15]. In addition, the periventricular and subcortical distribution of white matter lesions could interrupt the descending motor fibers arising from medial cortical areas, which are important for lower extremity motor control [16]. However, while the results of these neuroimaging studies contribute to our appreciation of the importance of brain structure to physiological falls risk, they do not provide specific guidance for refining or developing falls prevention strategies because white matter lesions are not currently modifiable once they present. Studies have also demonstrated the contribution of brain volume to physiological falls risk. Specifically, reduced grey matter volume within sensorimotor and frontal parietal regions of the brain is associated with both reduced gait speed and impaired balance [17,18].

Of particular relevance to falls prevention, targeted exercise training is beneficial for both brain volume, as assessed by MRI, and brain function, as assessed by fMRI [19]. What has not been well examined to date is the contribution of brain function to physiological falls risk. Using functional magnetic resonance imaging (fMRI), we previously demonstrated that reduced activity in the posterior lobe of the right cerebellum during an executive-challenging cognitive task may be an underlying neural mechanism for increased falls risk [20].

To our knowledge, it is currently unknown whether the function of brain regions responsible for executive functions are independently associated with *reduced *physiological falls risk after accounting for relevant factors such as baseline age, baseline physiological falls risk, and baseline brain volume. Yet, such knowledge would facilitate the development and refinement of targeted interventions to reduce physiological falls risk in older adults. Thus, we used fMRI to examine the functional neural correlates of executive functioning that are independently associated with reduced physiological falls risk among community-dwelling senior women.

## Methods

### Participants

The sample for this analysis consisted of a subset of 155 women who consented and completed a 12-month randomized controlled trial of exercise (NCT00426881) that primarily aimed to examine the effect of once-weekly or twice-weekly resistance training compared with a twice-weekly balance and tone exercise intervention on cognitive performance of executive functions. The design and the primary results of the study have been reported elsewhere [21].

We recruited and randomized 155 senior women who: 1) were aged 65-75 years; 2) were living independently in their own home; 3) obtained a score ≥ 24 on the Mini-Mental Status Examination (MMSE) [22]; and 4) had a visual acuity of at least 20/40, with or without corrective lenses. We excluded those who: 1) had a diagnosed neurodegenerative disease (e.g., AD) and/or stroke; 2) were taking psychotropic drugs; 3) did not speak and understand English; 4) had moderate to significant impairment with ADLs as determined by interview; 5) were taking cholinesterase inhibitors within the last 12 months; 6) were taking anti-depressants within the last six months; or 7) were on oestrogen replacement therapy within the last 12 months.

Ethical approval was obtained from the Vancouver Coastal Health Research Institute (V06-0326) and the University of British Columbia's Clinical Research Ethics Board (H06-0326). All participants provided written informed consent.

### Randomization

The randomization sequence was generated by http://www.randomization.com and was concealed until interventions were assigned. This sequence was held independently and remotely by the Research Coordinator. Participants were enrolled and randomised by the Research Coordinator to one of three groups: once-weekly resistance training (1x RT), twice-weekly resistance training (2x RT), or twice-weekly balance and tone (BAT).

### Exercise Intervention

#### Resistance Training

All classes were 60 minutes in duration. The protocol for this program was progressive and high-intensity in nature. Both a Keiser^® ^Pressurized Air system and free weights were used to provide the training stimulus. Other key strength exercises included mini-squats, mini-lunges, and lunge walks.

#### Balance and Tone

This program consisted of stretching exercises, range of motion exercises, kegals, balance exercises, and relaxation techniques. This group served to control for confounding variables such as physical training received by traveling to the training centres, social interaction, and lifestyle changes secondary to study participation.

### Descriptive Variables

Global cognition was assessed using the MMSE [22]. We used the 15-item Geriatric Depression Scale (GDS) [23] to screen for depression. Functional Comorbidity Index was calculated to estimate the degree of comorbidity associated with physical functioning [24]. This scale's score is the total number of comorbidities.

### Dependent Variable: Physiological Falls Risk

Physiological falls risk was assessed using the short form of the physiological profile assessment (PPA; Prince of Wales Medical Research Institute, AUS) to assess physiological falls risk. The PPA measures five domains of physiological functioning - dominant hand reaction time, postural sway, contrast sensitivity, proprioception, and dominant quadriceps strength - and computes a global falls risk score that has 75% accuracy for predicting falls. Global PPA scores < 0 indicate low falls risk, 0 to 1 indicate mild falls risk, 1 to 2 indicate moderate falls risk, and scores > 2 indicate high falls risk. We calculated change in physiological falls risk as the difference score between the baseline global PPA score and the trial completion PPA score; higher PPA change scores indicate greater reductions in physiological falls risk.

### Independent Variables of Interest

#### Brain Structure: Anatomical MRI

Baseline brain volume was measured via high-resolution, T1-weighted structural MRI images obtained using a Philips Achieva 3T scanner (TR = 8 ms, TE = 3.7 ms, bandwidth = 2.26 kHz, voxel size = 1 × 1 × 1 mm). Brain tissue volume, normalized for subject head size, was estimated with SIENAX [25], part of FSL (FMRIB's Software Library, Version 4.1.4) [26]. SIENAX starts by extracting brain and skull images from the single whole-head T1 image [27]. The brain image was then affine-registered to Montreal Neurological Institute (MNI) 152 space [28,29]. Next, tissue-type segmentation with partial volume estimation was carried out [30] in order to calculate baseline total volume of brain tissue, total white matter volume, and total grey matter volume.

#### Brain Function: Functional MRI

Transverse echo-planar imaging (EPI) images in-plane with the AC-PC line were acquired using a gradient-echo pulse sequence and sequential slice acquisition (TR = 2000 ms, TE = 30 ms, flip angle = 90°, 36 contiguous slices at 3 mm skip 1 mm, in-plane resolution of 128 × 128 pixels reconstructed in a FOV of 240 mm). Each functional run began with four TR's during which no data were acquired to allow for steady-state tissue magnetization. A total of 148 EPI volumes were collected in each functional run, and a total of 6 functional runs were collected for each participant.

**Figure 1:**
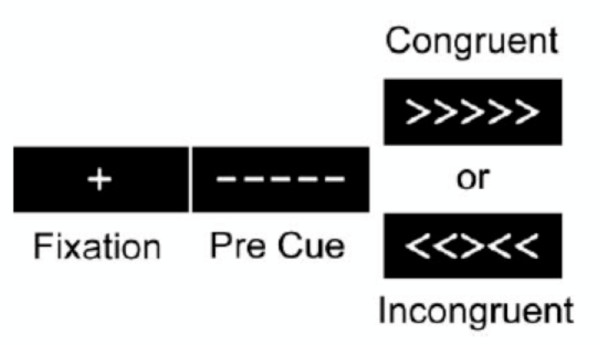
**The Flanker Task**. Participants were presented with a 13.5-sec fixation cross, which was followed by a 500 milliseconds pre-cue that informed participants that the critical stimulus will appear soon. Finally, an array of five arrows was on the screen. Participants responded to the orientation of the central arrow cue by pressing a button with their left hand if the central arrow cue pointed to the left and with their right hand if the central arrow cue pointed to the right. During one half of the trials, the flanking arrows faced in the same direction as the central arrow cue (i.e., congruent trials), and during the other half, they pointed in the opposite direction as the central arrow cue (i.e., incongruent trials). These stimuli remained on the screen for 2,000 milliseconds. Each participant underwent six successive five-minute blocks where they were presented with 17 trials that are first-order counterbalanced such that consistent and inconsistent trials followed each other equally [31]. This paradigm is sensitive to age-related decrements in attention control [48].

During scanning, participants performed a modified Eriksen flanker task [31] -- a task that engages the executive cognitive processes of selective attention and conflict resolution (Figure 1). Participants viewed displays with an arrow at central fixation, flanked by a pair of arrows on either side. In half the trials, the flanking arrows pointed in the same direction as the central arrow cue (e.g., < < < < <; congruent condition), and in the other half, the flanking arrows pointed in the opposite direction (e.g., > > < > >; incongruent condition). There were four event types based on whether the central arrow was congruent versus incongruent with the distracter arrows and whether it pointed to the left or right. A central fixation cross was presented for 500 milliseconds at the beginning of each trial. The target stimulus (arrows) was then shown for 2000 milliseconds. An average of 13500 milliseconds of blank screen was presented between each trial, jittered between 11500 and 15000 milliseconds. Each participant underwent six successive five-minute blocks where they were presented with 17 trials that were first-order counterbalanced such that congruent and incongruent trials followed each other equally. The participants' task on each trial was to signal the direction the central arrow points via a simple key press. Reaction time was recorded in milliseconds. At the end of the sessions, a high-resolution scan allowed each participant's anatomical and functional images to be co-registered during data analysis.

Functional MRI data were processed and analyzed using SPM2 (http://www.fil.ion.ucl.ac.uk/spm). For each participant, the EPI images were corrected for motion using the INRIalign toolbox for SPM2 (http://www-sop.inria.fr/epidaure/software/INRIAlign/). The resulting images were spatially-normalized into MNI stereotaxic coordinates using the EPI template provided with SPM2 [32], and spatially smoothed using an isotropic 8 mm Gaussian kernel. For each participant, the smoothed, normalized EPI data were analyzed via multiple regression using a fixed-effects general linear model [33]. In particular, the event-related responses to the onsets of the stimuli was examined, with each participant's model including four event-related regressors: 1) one for each combination of target type (i.e., left or right); 2) and distracter condition (i.e., congruent or incongruent). Regressors were based on the canonical event-related hemodynamic response function, temporal derivatives of the event-related responses were included as additional regressors, and low-frequency scanner and/or physiological noise was modeled via linear, quadratic, and cubic regressors of non-interest. Group-level analyses were then based on a random-effects model using one-sample t-tests, with a threshold of *p *< 0.05, corrected, and a minimum extent threshold of 10 contiguous voxels. Mean beta values reported for clusters identified in the group-level data were extracted from the SPM2 data files using custom scripts implemented in MATLAB (The MATHWORKS Inc., Natick, MA). The group-level cluster means were calculated by first determining each participant's mean beta across all voxels in the given cluster. All reported voxel coordinates were converted to Talairaich coordinates [34] using the mni2tal MATLAB script (http://imaging.mrc-cbu.cam.ac.uk/imaging/MniTalairach). The mean beta values were then imported to SPSS.

### Statistical Analyses

Descriptive data are reported for variables of interest. Data were analyzed using SPSS Windows Version 18.0 (SPSS Inc., Chicago, IL). The associations between the variables were determined using the Pearson product moment coefficient of correlation.

A multiple linear regression model was constructed to determine the independent association of the neural correlates of executive functioning, as assessed by fMRI, with change in physiological falls risk over the 12-month intervention study, as assessed by PPA. Baseline age, experimental group, and baseline physiological falls risk were statistically controlled by entering these three variables into the regression model first. These independent variables were determined from the results of the Pearson product moment coefficient of correlation analyses (i.e., baseline PPA score) and based on biological relevance, such as experimental group and age.

Baseline total brain volume, total white matter volume, and total grey matter volume were then entered into regression model and only those that significantly improved the model were included (i.e., stepwise). Finally, regions of the brain (i.e., clusters) showing increases in the hemodynamic response on incongruent relative to congruent trials of the flanker task were then entered into the model using a stepwise approach. Alpha was set at *p *≤ 0.05.

## Results

### Participants and Variables of Interest

Of the 155 participants who consented and were randomized at baseline, 135 completed the 12-month trial. Seventy-three of the 135 participants consented and completed baseline MRI and fMRI scanning.

Table 1 reports the baseline descriptive statistics for this cohort. The mean baseline PPA score was 0.10, indicating mild falls risk. At the end of the 12-month trial, the 73 women demonstrated a mean change of 0.10 in the PPA score. A paired t-test indicated that this was not a statistically significant change (*p *= 0.06).

**Table 1:** Descriptive statistics for variables of interest (N = 73).

Variable ^1 ^	BAT (n = 22) Mean (SD)	1x RT (n = 28) Mean (SD)	2x RT (n = 23) Mean (SD)	Total (N = 73) Mean (SD)
Age (yr)	69.6 (3.1)	69.5 (2.7)	69.1 (3.1)	69.4 (2.9)

Height (cm)	161.5 (6.2)	162.0 (7.5)	162.4 (6.9)	161.9 (6.9)

Weight (kg)	67.1 (10.9)	67.9 (13.6)	68.6 (13.0)	67.9 (12.5)

Education				

Less than Grade 9 ^2^	0.0 (0.0)	0.0 (0.0)	0.0 (0.0)	0.0 (0.0)

Grade 9 to 12 without Certificate or Diploma ^2^	2.0 (9.1)	2.0 (7.1)	0.0 (0.0)	4.0 (5.5)

High School Certificate or Diploma ^2^	5.0 (22.7)	3.0 (10.7)	5.0 (21.7)	13.0 (17.8)

Trades or Professional Certificate or Diploma ^2^	3.0 (13.6)	6.0 (21.4)	2.0 (8.7)	11.0 (15.1)

University Certificate or Diploma ^2^	4.0 (18.2)	5.0 (17.9)	4.0 (17.4)	13.0 (17.8)

University Degree ^2^	8.0 (36.4)	12.0 (42.9)	12.0 (52.2)	32.0 (43.8)

MMSE Score (max. 30 pts)	28.8 (1.3)	28.6 (1.3)	28.8 (1.0)	28.7 (1.2)

Falls in the Last 12 Months (yes/no) ^2^	8 (36.4)	7 (25.0)	9 (39.1)	24 (32.9)

Geriatric Depression Scale (/15 pts)	0.7 (2.2)	0.1 (0.8)	0.6 (1.6)	0.5 (1.6)

Functional Comorbidity Index (/18 pts)	2.2 (1.3)	1.9 (1.7)	1.7 (1.5)	1.9 (1.5)

Baseline Physiological Profile Assessment Score	0.10 (0.91)	0.06 (0.89)	0.16 (1.11)	0.10 (0.96)

Total Brain Volume ^3^	1404767.07 (61101.38)	1392824.85 (74770.29)	1425571.35 (53607.47)	1406741.26 (65216.88)

White Matter Volume ^3^	673259.09 (37763.87)	668611.61 (33667.89)	680775.90 (30457.92)	673844.81 (33920.23)

Gray Matter Volume ^3^	731508.20 (30004.57)	731957.93 (35834.91)	746535.05 (35339.77)	736415.19 (34256.96)

Change in Physiological Falls Risk	0.25 (0.97)	0.04 (0.88)	0.34 (0.82)	0.10 (0.96)

^1 ^BAT = Balance and Tone; 1x RT = once-weekly resistance training; 2x RT = twice-weekly resistance training; yr = year; kg = kilogram; MMSE = Mini-Mental State Examination; sec = seconds.

^2 ^Count = number of "yes" cases within each group. % = percent of "yes" within each group.

^3 ^Brain volume = mm^3^.

Behavioural performance on the flanker task was calculated as percent increase in reaction time to incongruent stimuli, over and above the average reaction time to congruent stimuli {[(incongruent reaction time - congruent reaction time)/congruent reaction time] × 100} [31]. The percent increase measure is derived to reflect interference unbiased by differences in base reaction time. Only correct responses were included in the analysis. Mean interference score for BAT, RT1, and RT2 were 16.59 (SD = 13.07), 19.92 (SD = 2.52), and 27.98 (SD = 13.77), respectively.

**Figure 2:**
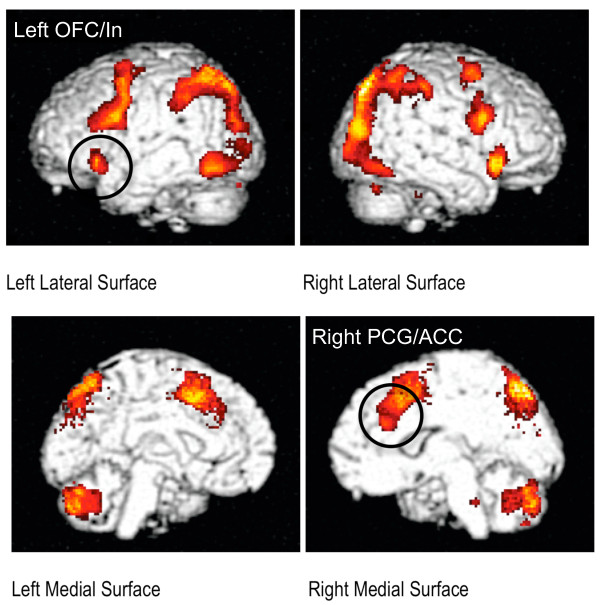
**Brain Regions Demonstrating an Increased Hemodynamic Response on Incongruent Relative to Congruent Trials**. Data are group-averaged across all 83 participants and shown on a rendered brain provided with SPM2. Data were thresholded at ***P ***< 0.05 (corrected) and a minimum cluster size of 10 contiguous voxels. The left OFC/In and right PCG/ACC both contributed significantly to our model predicting change in physiological falls risk.

Consistent with previous studies using the flanker task, regions showing increases in the hemodynamic response on incongruent relative to congruent trials included bilateral inferior and middle frontal gyri, frontal orbital cortex, anterior cingulate cortex (ACC), bilateral precuneus, and the right cerebellum (Figure 2); 14 clusters were identified (Table 2).

**Table 2:** Voxel Cluster Statistics from fMRI.

Hemisphere	Structure	BA ^1^	K ^2^	t ^3^	MNI			TAL		
					**X**	**Y**	**Z**	**X**	**Y**	**Z**

Right	Lateral occipital cortex	19	4247	9.51	28	-78	42	28	-74	42

Right	Frontal orbital cortex	47	597	8.18	36	24	-4	36	23	-5

Right	Posterior cerebellum		1131	7.95	8	-80	-34	8	-79	-25

Left	Lateral occipital cortex	37	626	7.67	-48	-70	-12	-48	-68	-7

Right	Paracingulate gyrus	32	1634	7.49	8	20	44	8	21	39

Left	Lateral occipital cortex	7	2915	7.43	-22	-72	32	-22	-68	33

Left	Middle frontal gyrus	6	1620	7.20	-26	0	50	-26	2	46

Right	Middle frontal gyrus	6	631	7.03	26	2	48	26	4	44

Right	Inferior frontal gyrus	9	699	6.97	54	14	28	53	15	25

Left	Frontal orbital cortex	47	198	6.51	-32	24	-6	-32	23	-6

Left	Frontal orbital cortex	47	122	6.19	-46	20	-10	-46	19	-9

Right	Supramarginal gyrus	40	82	6.04	28	-68	-28	28	-67	-20

Right	Posterior cerebellum		25	5.72	14	-76	-50	14	-76	-38

Right	Anterior cerebellum		21	5.69	16	-38	-34	16	-38	-27

Reported coordinates and t values are for the cluster maxima.^1 ^BA = Brodmann's area. ^2 ^K = # of voxels in the cluster. ^3 ^All *p *values < 0.05.

### Correlation Coefficients

Table 3 reports the bivariate correlation coefficients of those variables included in the final multiple linear regression model. Baseline physiological falls risk was positively and significantly associated with change in physiological falls risk (*p *< 0.001). Baseline total brain volume, total white matter volume, and activation (i.e., hemodynamic response) in the left frontal orbital cortex extending towards the insula (OFC/In) were negatively and significantly associated with change in physiological falls risk (*p *< 0.05). In our bivariate analysis, age, experimental group, and activation in the right paracingulate gyrus extending towards the anterior cingulate cortex (PCG/ACC) were not associated with change in physiological falls risk (*p *> 0.26).

**Table 3:** Multiple linear regression model summary for improved physiological falls risk.

	Δ PPA Score (Baseline Score - Trial Completion Score)					
	
Independent Variable	r	R^2^	R^2 ^Change	Unstandardized B (Standard Error)	Standardized *β*	*p *- value
**Model 1**	0.565	0.319	0.319			
Group	0.040			0.015 (0.112)	0.013	0.896
Age	-0.078			-0.064 (0.031)	-0.211	0.043
Baseline PPA Score	0.526**			0.529 (0.094)	0.575	<0.001
**Model 2**	0.619	0.383	0.064			
Group	0.040			0.040 (0.107)	0.035	0.713
Age	-0.078			-0.068 (0.030)	-0.224	0.026
Baseline PPA Score	0.526**			0.521 (0.090)	0.566	<0.001
White Matter Volume	-0.263*			-6.670E-6 (0.000)	-0.255	0.010
**Model 3**	0.698	0.487	0.104			
Group	0.040			0.034 (0.099)	0.030	0.733
Age	-0.078			-0.088 (0.028)	-0.287	0.003
Baseline PPA Score	0.526**			0.504 (0.083)	0.548	<0.001
White Matter Volume	-0.263*			-8.800E-6 (0.000)	-0.337	<0.001
Cluster 3 ^1^	-0.258			-0.654 (0.177)	-0.339	0.014
**Model 4**	0.729	0.531	0.044			
Group	0.040			0.023 (0.095)	0.021	0.809
Age	-0.078			-0.087 (0.027)	-0.286	0.002
Baseline PPA Score	0.526**			0.474 (0.081)	0.515	<0.001
White Matter Volume	-0.263*			-1.000E-5 (0.000)	-0.383	<0.001
Cluster 3 ^1^	-0.258*			-1.159 (0.266)	-0.601	<0.001
Cluster 7 ^2^	-0.055			0.637 (0.271)	0.329	0.016

* ***P ***≤ 0.05

** ***P ***≤ 0.001

^1 ^Cluster 3 is the region of left frontal orbital cortex extending towards the insula.

^2 ^Cluster 7 is the region of right paracingulate gyrus extending towards the anterior cingulate cortex.

### Linear Regression Model

Baseline age, experimental group, and baseline physiological falls risk, accounted for 31.9% of the variance in change in physiological falls risk (Table 3). Adding baseline total white matter volume resulted in an R-square change of 6.4% and significantly improved the regression model (*F *Change = 7.1, *p *= 0.01). Adding activation in the left OFC/In to the model resulted in an R-square change of 10.4% and significantly improved the model (*F *Change = 13.6, *p *< 0.001). Finally, the inclusion of activation in the right PCG/ACC resulted in significant R-square change of 4.4% (*F *Change = 6.6, *p *= 0.02). The total variance accounted by the final model was 53.1% (Table 3). Based on the standardized betas, the left OFC/In was most associated with reduced physiological falls risk.

## Discussion

Recent evidence strongly suggests that changes in brain structure with age contribute to problems with mobility [35-39]. However, less is known about the role of brain function [20]. To our knowledge, our study is the first to demonstrate the independent contribution of brain function to reduced physiological falls risk among community-dwelling seniors. Specifically, after accounting for baseline age, experimental group, baseline physiological falls risk, and baseline total white matter volume, activation in the left OFC/In was negatively and independently associated with reduced physiological falls risk in community-dwelling senior women over a 12-month period. In contrast, activation in the PCG/ACC was positively and independently associated with reduced physiological falls risk.

The two regions included in our multiple linear regression model -- the left OFC/In and the right PCG/ACC -- are both part of the neural network associated with response inhibition and selective attention [40-43]. Response inhibition - the ability to avoid unwanted, inappropriate responses - is associated with falls in seniors. For example, Anstey and colleagues [44] reported that senior fallers (both single and recurrent) performed significantly worse on a measure of response inhibition compared to non-fallers. The authors suggested that reduced inhibition results from age-related declines in functioning of the prefrontal cortex, which contributes to falls. Given that movement through the environment requires attending to relevant stimuli and inhibiting prepotent, yet potentially unsafe, responses, it is not surprising that brain regions associated with response inhibition and selective attention are related to falls risk.

Importantly, we found that activation in the left OFC/In was negatively associated with reduced physiological falls risk, whereas activation in the PCG/ACC was positively associated with reduced physiological falls risk. Increased activation in the frontal cortex during an executive task, such as the flanker, is associated with better task performance [31]. In contrast, increased activation of the anterior cingulate cortex in older adults is associated with reduced task performance [31]. In particular, increased anterior cingulate cortex activation is hypothesized to be an indicator of greater cognitive effort, such that the anterior cingulate cortex is less efficient at triggering the prefrontral system to engage cognitive control [45].

Our volumetric brain results also suggest that total white matter volume, rather than total grey matter volume, is associated with change in physiological falls risk. Previous research suggests that white matter declines at a faster rate than grey matter in otherwise healthy older adults [46]. Our results extends this finding by suggesting the loss of total white matter volume may be an early indicator of increased falls risk among community-dwelling older adults.

Of particular clinical relevance, the results of our study suggest that individuals at higher risk for future falls have greater potential for risk reduction than those at lower risk for falls. Specifically, our multiple regression model showed that baseline physiological falls risk was positively associated with change in physiological falls risk. Hence, our current study results concurs and extends that of a previous meta-analysis that concluded exercise-based falls prevention strategies are most effective among those at the greatest risk [47]. This suggests that one intervention strategy for falls prevention may be to target those who are at greatest risk for falls.

We note that of the independent variables included in our regression model, baseline activation of the left OFC/In was most associated with reduced physiological falls risk. Hence, while many falls interventions focus on balance training, our study suggests that future falls prevention strategies should potentially incorporate intervention components that induce neurocognitive plasticity (i.e., changes in brain function). Future work is needed to establish whether such interventions would be effective. Current evidence suggests that targeted aerobic exercise training has specific benefits on neurocognitive plasticity in brain regions that are responsible for selective attention and response inhibition [31]. Therefore, promoting plasticity in brain regions associated with these key executive functions may have a positive impact on falls prevention.

We acknowledge that our finding that a negative association between baseline total white matter volume and change in physiological falls risk is significantly associated with reduced falls risk contrasts previous cross-sectional studies on gray matter volume, balance, and mobility. Specifically, Rosano and colleagues [17,18] found that reduced gait speed and impaired balance - key risk factors for falls -- were significantly correlated with reduced grey matter volume within sensorimotor and frontal parietal regions in the brain. However, we highlight that our study examined the independent contribution of baseline volumetric brain measures to ***change ***in falls risk (i.e., longitudinal study design versus cross-sectional design) and hence our conclusion that those at the greater risk for future falls (i.e., smaller baseline total white volume) have greater potential for falls risk reduction (i.e., greater change in PPA scores).

We recognize the limitations of our study. A key limitation is that we did not quantify white matter lesions within total white matter volume. We note that our study sample consisted exclusively of independent community-dwelling senior women who were without significant physical and cognitive impairments and without a significant history of falls. Thus, the results of our study may not generalize beyond this population of senior women and we may have underestimated the contribution of brain function to change in physiological falls risk. Future prospective studies are needed to test whether the present findings also apply to larger, more heterogeneous populations.

To conclude, the function of brain regions underlying response inhibition and selective attention was independently associated with reduced physiological falls risk. Hence, future falls prevention strategies should potentially incorporate intervention components, such as aerobic exercise training, that induce neurocognitive plasticity in the neural network that supports response inhibition and selective attention.

## Declaration of Competing interests

The authors declare that they have no competing interests.

## Authors' contributions

TLA conceived and designed the study, acquired data, and analyzed and interpreted the data. LSN and CLH acquired data and participated in the statistical analysis. TLA, LSN, CLH, and TCH drafted and revised the manuscript. All authors read and approved the final manuscript.
